# Rituximab and improved nodular regenerative hyperplasia-associated non-cirrhotic liver disease in common variable immunodeficiency: a case report and literature study

**DOI:** 10.3389/fimmu.2023.1264482

**Published:** 2023-09-19

**Authors:** Willem Roosens, Frederik Staels, Sien Van Loo, Stephanie Humblet-Baron, Isabelle Meyts, Hadewijch De Samblanx, Chris Verslype, Hannah van Malenstein, Schalk van der Merwe, Wim Laleman, Rik Schrijvers

**Affiliations:** ^1^ Department of Microbiology, Immunology and Transplantation, Allergy and Clinical Immunology Research Group, KU Leuven, Leuven, Belgium; ^2^ Center for Human Genetics, University Hospitals Leuven, Leuven, Belgium; ^3^ Department of Microbiology, Immunology and Transplantation, Laboratory of Adaptive Immunology, KU Leuven, Leuven, Belgium; ^4^ Department of Microbiology, Immunology and Transplantation Laboratory of Inborn Errors of Immunity, KU Leuven, Leuven, Belgium; ^5^ Department of Pediatrics, University Hospitals Leuven, Leuven, Belgium; ^6^ Department of Hematology, Ziekenhuis Geel, Geel, Belgium; ^7^ Department of Gastroenterology and Hepatology, Section of Liver and Biliopancreatic disorders, University Hospitals Leuven, Leuven, Belgium; ^8^ Department of Chronic Diseases, Metabolism and Aging (CHROMETA), Laboratory of Hepatology, KU Leuven, Leuven, Belgium; ^9^ Medizinische Klinik B, Universitätsklinikum Münster, Münster University, Münster, Germany

**Keywords:** non-infectious complications, nodular regenerative hyperplasia, non-cirrhotic portal hypertension, rituximab, common variable immune deficiency (CVID), primary immunodeficiency, inborn errors of immunity

## Abstract

Common variable immunodeficiency (CVID) associated liver disease is an underrecognized and poorly studied non-infectious complication that lacks an established treatment. We describe a CVID patient with severe multiorgan complications, including non-cirrhotic portal hypertension secondary to nodular regenerative hyperplasia leading to diuretic-refractory ascites. Remarkably, treatment with rituximab, administered for concomitant immune thrombocytopenia, resulted in the complete and sustained resolution of portal hypertension and ascites. Our case, complemented with a literature review, suggests a beneficial effect of rituximab that warrants further research.

## 1 Introduction

Common variable immunodeficiency (CVID) is the most common primary immunodeficiency in adulthood and is characterized by a defective B cell differentiation, impaired antibody secretion and recurrent bacterial infections. More than 30% of patients also suffer from non-infectious comorbidities attributed to immune dysregulation. These have become the leading cause of death in CVID since immunoglobulin replacement therapy is insufficient to prevent or control their complications. Liver involvement is common in CVID, with a prevalence ranging from 9.3 to 79% in different cohort studies, and typically manifests with histologic nodular regenerative hyperplasia (NRH) (1). This porto-sinusoidal vascular disorder, associated with a range of other chronic inflammatory or infectious diseases, can lead to non-cirrhotic portal hypertension (NCPH) or liver cirrhosis, conferring a poor prognosis in these patients (2). The association with other inflammatory manifestations in CVID supports a shared immunopathogenesis, marked by increased autoreactive B cells and reduced T regulatory cells (3). Although these observations support the use of B and/or T cell modulating therapies, there is currently no established treatment to cure or prevent progression of NCPH in CVID. Commonly accepted supportive treatment for complicated cirrhosis and portal hypertension, including transjugular intrahepatic portosystemic shunt (TIPS), has been applied in CVID. Liver transplantation for CVID-associated end-stage liver disease has also been reported but is associated with a poor outcome due to disease recurrence (2). Etiological treatment is unknown. Oral budesonide has been shown to improve liver enzyme alterations in a single case with CVID-related NRH without portal hypertension (4). Systemic corticosteroids and anti-TNF treatment have been reported in cases with autoimmune hepatitis-like or granulomatous liver disease, with variable success (5). Rituximab, a chimeric monoclonal antibody targeted against the B cell marker CD20, is routinely used for the management of immune cytopenias and limited observational data support its use in CVID-related granulomatous and lymphocytic interstitial lung diseases (GLILD) (6). The effect of rituximab on CVID-related liver involvement has not been studied as such.

## 2 Case report

**Figure 1:**
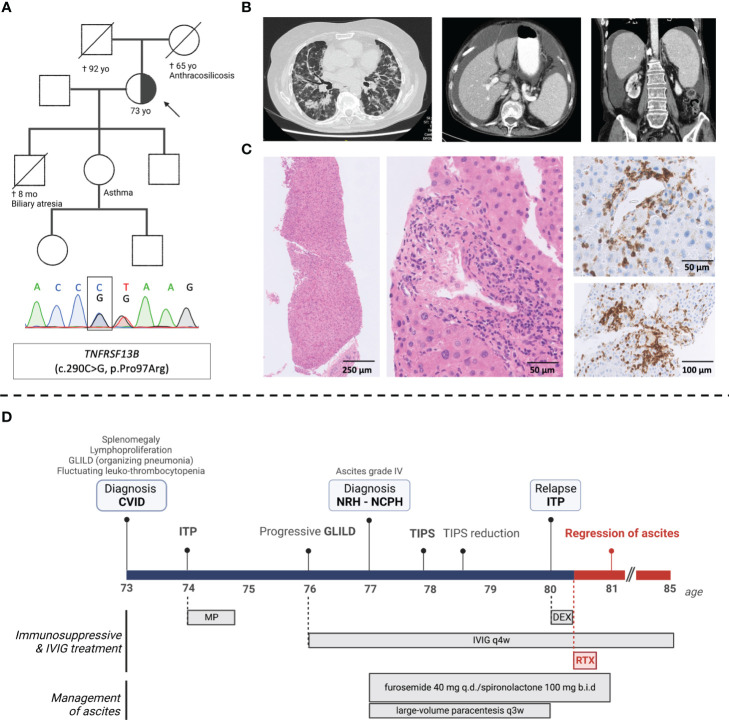
Clinical, genetic, histological, and radiological findings in a patient with late-onset CVID and severe multi-organ immune dysregulation. **(A)** Pedigree with index patient (arrow) (family members not sequenced), Sanger confirmation of the *TNFRSF13B* variant (c.290C>G); **(B)** computed tomography (CT) images showing diffuse peribronchial and bronchiolar glass ground-glass opacities and traction bronchiectasis; splenomegaly (17 cm diameter) and mild hepatomegaly with regular liver contours and umbilical vein recanalization; **(C)** (*from left-to-right)* liver biopsy showing subtle nodularity of liver parenchyma stained with H&E; lobular and periportal mononuclear infiltrates (H&E) predominated by CD3^+^ T-cells (immunohistochemical CD3 staining); **(D)** Timeline of the patient’s clinical course including clinical events and medication. CVID, common variable immunodeficiency*;* ITP, immune thrombocytopenia*;* IVIG, intravenous immunoglobulins; GLILD, granulomatous lymphocytic interstitial lung disease; TIPS, transjugular intrahepatic portosystemic shunt; NRH, Nodular regenerative hyperplasia; NCPH, Non-cirrhotic portal hypertension; TIPS, transjugular intrahepatic portosytemic shunt; MP, methylprednisolone; DEX, dexamethasone; RTX, rituximab.

We describe a 77-year-old female patient with late-onset CVID and severe multi-organ immune dysregulation, including splenomegaly, polyclonal lymphoproliferation, GLILD, and recurrent leuko- and thrombocytopenia (**Figure 1**). Panel-based exome sequencing revealed a heterozygous variant in *TNFRSF13B* (c.290C>G, p.Pro97Arg), encoding the transmembrane activator and calcium modulator and cyclophilin ligand interactor (TACI), a crucial regulator of B cell receptor activation. TACI mutations are frequently identified in CVID patients, and monoallelic deleterious variants favor the development of autoimmune disease (3), presumably by impairing central B cell tolerance whilst allowing for a residual B cell responsiveness.

New onset ascites (grade 3) led to a diagnosis of NCPH secondary to histologically proven NRH (**Figure 1C**). Treatment included a transjugular intrahepatic portosystemic shunt (TIPS), which was later reduced due to hepatic encephalopathy, large volume paracenteses every 3 to 4 weeks, and diuretics (spironolactone 200 mg and furosemide 40 mg daily). Three years later, after receiving rituximab (375 mg/m^2^ weekly for 4 weeks) preceded by a short course of dexamethasone (20 mg daily for 4 days) for immune thrombocytopenia relapse, a complete regression of ascites was noted, and diuretic treatment was discontinued. Platelet counts were quasi-normalized and remained steady after a single cycle of rituximab. Pulmonary findings and patient-reported exercise capacity were stable over a 5-year follow-up period, yet without significant radiological or spirometric improvement – possibly due to longstanding fibrotic lung disease, considering the late age at diagnosis (**Figure E1**).

Although (non-)invasive liver assessments were not repeated, the radiological disappearance of ascites up to 5 years post-treatment indicates a sustained decrease in portal hypertension.

## 3 Literature review and discussion

By reviewing the existing literature, we identified 119 CVID patients treated with rituximab either alone or in combination with corticosteroids or anti-metabolites, for non-malignant immune-mediated complications (**Table E1**). Liver disease, of any kind, was reported in 18 patients, but a response to rituximab was only mentioned in 4 of these cases, all with favorable outcome (**Table 1**). Overall, rituximab was reported to be safe and well tolerated, as in our patient.

**Table 1:** Reported effect on liver disease in CVID patients treated with rituximab for other indications.

Reference	Králíčková, P. et al. (7)	Pathria, M. et al. (8)	Boursiquot, J. N et al. (9)
*Article Type*	Abstract/Case Report	Case Report	Retrospective cohort
*Year of publication*	2018	2016	2013
*Total number of patients treated with RTX*	**n = 1**	**n = 1**	**n = 3**
*Indication for RTX*	GLILD	GLILD	GLILD
*RTX regimen*	375 mg/m^2^ IV weekly × 4, 6-monthly	375 mg/m^2^ IV weekly × 4, 6-monthly	375 mg/m^2^ IV weekly × 4
*Concomittant immune suppresive drugs*	Not reported	Azathioprine 1.5 mg/kg	Corticosteroids (n = 1)
*Reported liver disease*	Not reported	**Portal hypertension**	**Liver granulomatous disease** (n = 2)
*Effect on liver disease*	**Reduction in ALP**	**Resolution of ascites**	**Partial response** (n =1) and **complete response** (n=1)

A summary of the literature search, including the search strategy, is provided in the Supplementary Material (**Table E1**). GLILD, granulomatous lymphocytic interstitial lung disease; RTX, rituximab; ALP, alkaline phosphatase.

Liver involvement in CVID is heterogenous and different phenotypes may rely on a different pathogenetic mechanisms, including immune dysregulation, autoreactive antibodies, infections and microbial translocation. Nevertheless, NRH is the most common histological pattern observed in the liver of CVID patients with chronic cholestasis, NCPH, or liver cirrhosis, with or without granulomatous disease (1). Notably, cytotoxic T cells often predominate the intrasinusoidal inflammatory infiltrate, supporting a central role for T cell dysregulation as suggested in CVID-associated immune dysregulation (1, 3, 5). The effect of B cell depletion following rituximab could be explained by an indirect restoration of T helper cell type 1 (Th1)/Th2 ratio and the increase of regulatory T cells, as reported in other autoimmune conditions (6).

Taken together, NRH is likely to represent an immune-mediated manifestation of CVID and the findings reported here support a potential beneficial effect of rituximab on hepatic disease course. This would be in line with the effect of rituximab in other non-infectious CVID comorbidities such as autoimmune cytopenia and GLILD. However, we should be aware of a potential reporting bias. Therefore, future prospective studies are warranted to more systematically study the effect of rituximab on CVID-associated liver disease.

## Data availability statement

The original contributions presented in the study are included in the article/**Supplementary Materials**, further inquiries can be directed to the corresponding author/s.

## Ethics statement

The studies involving humans were approved by The Ethics Committee Research UZ/KU Leuven. The studies were conducted in accordance with the local legislation and institutional requirements. The participants provided their written informed consent to participate in this study. Written informed consent was obtained from the individual(s) for the publication of any potentially identifiable images or data included in this article.

## Author contributions

WR: Conceptualization, Data curation, Formal Analysis, Funding acquisition, Methodology, Writing – original draft, Writing – review & editing, Investigation, Validation. FS: Conceptualization, Funding acquisition, Supervision, Writing – review & editing. SV: Data curation, Investigation, Methodology, Software, Visualization, Writing – review & editing. SH: Investigation, Supervision, Writing – review & editing. IM: Supervision, Writing – review & editing, Resources. HD: Data curation, Formal Analysis, Investigation, Writing – review & editing. CV: Investigation, Writing – review & editing. Hv: Investigation, Writing – review & editing. SM: Investigation, Writing – review & editing. WL: Investigation, Writing – review & editing. RS: Writing – original draft, Writing – review & editing, Resources, Supervision.

## Supplementary material

Supplementary Figure 1
**(A)** Main immunological and hepatic laboratory findings at diagnosis (positive anti-HBs antibodies possibly secondary to passive transfer with IVIG therapy); **(B)** results of invasive hepatic venous pressure gradient measurement (pre-TIPS) and periprocedural fluoroscopic image of transjugular intrahepatic portosystemic shunt (TIPS); **(C)** Sequential lung function testing (shown as percentage of predicted value), post-treatment testing is marked in blue. ALP, alkaline phosphatase; GGT, gamma-glutamyltransferase; ALT, alanine transaminase; AST, aspartate transaminase; LDH, lactate dehydrogenase; PT, prothrombin time; ANA, antinuclear antibody; AMA, antimitochondrial antibody; ASMA, anti-smooth muscle antibody; IGRA, interferon gamma release assay; HbsAb, hepatitis B surface antibody; FHVP, free hepatic venous pressure; WHVP, wedged hepatic venous pressure; HVPG, hepatic venous-portal gradient; FEV1, forced expiratory volume during the first second; FVC, forced vital capacity; RV, residual volume; TLC, total lung capacity; DLCO, diffusing capacity for carbon monoxide; KCo, carbon monoxide transfer coefficient.

Supplementary Table 1 Overview of articles reporting on the use of rituximab for the treatment of CVID-related inflammatory complications (in chronological order). For data collection, we performed a comprehensive literature search in the electronic database MEDLINE with the following search terms: “common variable immunodeficiency”, “CVID” and “rituximab”. Relevant references within the retrieved articles were evaluated and included if relevant. Red *=* articles reporting on liver involvement, Bold = articles reporting on the effect of rituximab on liver involvement. NA = not applicable or not reported, * unknown whether patients with reported liver disease were treated with rituximab.
