# Supplementary material for: Rituximab and improved nodular regenerative hyperplasia-associated non-cirrhotic liver disease in common variable immunodeficiency: a case report and literature study
Source: Front Immunol. 2023 Sep 19;14:1264482. doi: 10.3389/fimmu.2023.1264482 (PMC10546204; doi:10.3389/fimmu.2023.1264482)
Supplement: Supplementary Figure 1 — (A) Main immunological and hepatic laboratory findings at diagnosis (positive anti-HBs antibodies possibly secondary to passive transfer with IVIG therapy); (B) results of invasive hepatic venous pressure gradient measurement (pre-TIPS) and periprocedural fluoroscopic image of transjugular intrahepatic portosystemic shunt (TIPS); (C) Sequential lung function testing (shown as percentage of predicted value), post-treatment testing is marked in blue. ALP, alkaline phosphatase; GGT, gamma-glutamyltransferase; ALT, alanine transaminase; AST, aspartate transaminase; LDH, lactate dehydrogenase; PT, prothrombin time; ANA, antinuclear antibody; AMA, antimitochondrial antibody; ASMA, anti-smooth muscle antibody; IGRA, interferon gamma release assay; HbsAb, hepatitis B surface antibody; FHVP, free hepatic venous pressure; WHVP, wedged hepatic venous pressure; HVPG, hepatic venous-portal gradient; FEV1, forced expiratory volume during the first second; FVC, forced vital capacity; RV, residual volume; TLC, total lung capacity; DLCO, diffusing capacity for carbon monoxide; KCo, carbon monoxide transfer coefficient. [file DataSheet_1.docx]

**Online repository. Figure E1**


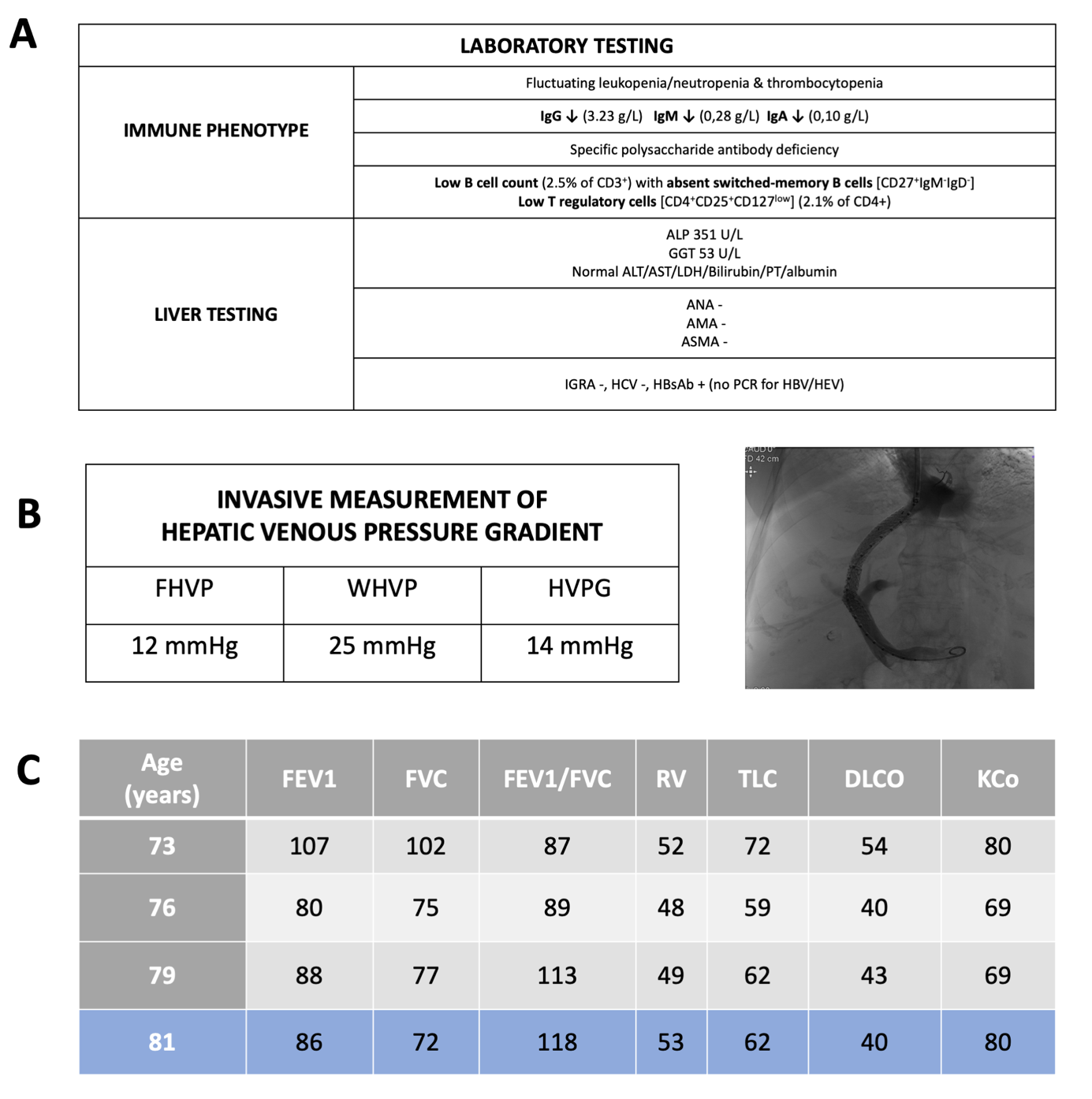


**A)** Main immunological and hepatic laboratory findings at diagnosis (positive anti-HBs antibodies possibly secondary to passive transfer with IVIG therapy); **B)** results of invasive hepatic venous pressure gradient measurement (pre-TIPS) and periprocedural fluoroscopic image of transjugular intrahepatic portosystemic shunt (TIPS); **C)** Sequential lung function testing (shown as percentage of predicted value), post-treatment testing is marked in blue.

*Abbrevations*: **ALP** = alkaline phosphatase, **GGT** = gamma-glutamyltransferase, **ALT** = alanine transaminase, **AST** = aspartate transaminase, **LDH** = lactate dehydrogenase, **PT**= prothrombin time,  **ANA** = antinuclear antibody, **AMA** = antimitochondrial antibody, **ASMA** = anti-smooth muscle antibody, **IGRA**= interferon gamma release assay, **HbsAb** = hepatitis B surface antibody, **FHVP** = free hepatic venous pressure, **WHVP** = wedged hepatic venous pressure, **HVPG** = hepatic venous-portal gradient, **FEV1** = forced expiratory volume during the first second, **FVC** = forced vital capacity, **RV** = residual volume, **TLC** = total lung capacity, **DLCO** = diffusing capacity for carbon monoxide, **KCo** = carbon monoxide transfer coefficient.

**Online repository, Table E1:** Overview of articles reporting on the use of rituximab for the treatment of CVID-related inflammatory complications (in chronological order)

|  | **Type of publication** | **Year** | **Total no. of patients** | **No. of patients treated with RTX** | **Indication for RTX** | **RTX regimen** | **Concomittant immune suppressants** | **RTX-related complications** | **Effect on comorbidities** | **Reported liver involvement** | **Reported effect on liver disease** | **Reference (PMID)** |
| --- | --- | --- | --- | --- | --- | --- | --- | --- | --- | --- | --- | --- |
| 1 | Case Report | 2005 | 1 | 1 | ITP | 375 mg/m^2^ IV weekly × 5 | dexamethasone 40mg 4d | / | + (ITP) | NRH with severe portal hypertension | NA | 16127007 |
| 2 | Case Report | 2006 | 1 | 1 | Autoimmune cytopenia | 375 mg/m^2^ IV weekly × 4 | Corticosteroids | / | + (autoimmune cytopenia) | NA | NA | 16823831 |
| 3 | Case Report | 2007 | 1 | 1 | Autoimmune cytopenia | 375 mg/m^2^ IV weekly, 1x | / | / | + (only ITP) | NA | NA | 17483248 |
| 4 | Case report | 2010 | 1 | 1 | ITP | 375 mg/m^2^ IV weekly × 4 | / | NA | + (ITP) | NA | NA | 20635793 |
| 5 | Retrospective multicentre study | 2011 | 33 | 33 | Autoimmune cytopenia | Several regimens | NA | Severe infections (n = 8) of whom 4 were not on IVIG/SCIG | + (autoimmune cytopenia) | Cholestatic hepatitis (n = 1) | NA | 21981575 |
| **6** | **Retrospective cohort** | **2013** | **59** | **3** | **GLILD** | **NA** | **Corticosteroids (n =1)** | **/** | **+ (GLILD, lymphadenopathies)** | **Granulomatous liver disease (n = 3/59)** | **Partial remission (n =1) and complete remission (n = 1 of liver granulomatous disease** | **22986767** |
| 7 | Retrospective multicentre study | 2013 | 45 | 5 | Autoimmune cytopenia | NA | corticosteroids, high-dose IVIG, splenectomy | NA | + (autoimmune cytopenia) | Undefined liver disease (n = 3/45*****) | NA | 23480186 |
| 8 | Case Report | 2013 | 1 | 1 | ITP | 375 mg/m^2^ IV weekly × 4 | no | Unmasking hypogammaglobulinemia with infections | + (ITP) | hepatosplenomegaly and hyperplasia on spleen biopsy | NA | 25213054 |
| 9 | Retrospective monocentre study | 2013 | 7 | 7 | GLILD | 375 mg/m^2^ IV weekly × 4 | AZA, 6-MP, or MMF | / | + (GLILD, reduction in spleen size) | NA | NA | 22930256 |
| 10 | Orginal article | 2014 | 6 | 1 | GLILD | 375 mg/m^2^ IV weekly | NA | / | + (GLILD) | NA | NA | 24131823 |
| 11 | Case Report | 2015 | 1 | 1 | GLILD, neuropthalmic sarcoidosis | 375 mg/m^2^ IV weekly × 4, 6-monthly | AZA, prednisone | / | + (GLILD, intracranial lesion) | NA | NA | 25609323 |
| **12** | **Case Report** | **2016** | **1** | **1** | **GLILD** | **375 mg/m^2^ IV weekly × 4, 4 to 6-monthly** | **IVIG, AZA** | **/** | **+ (GLILD, ITP)** | **Ascites (SAAG > 1.1)** | **Reduction of ascites** | **27335365** |
| 13 | Case Report | 2016 | 1 | 1 | ITP | NA | / | Severe bacterial infections (no IVIG/SCIG) | + (ITP) | NA | NA | 27276370 |
| 14 | Case Report | 2016 | 1 | 1 | Lymphoproliferative disease | 375 mg/m^2^ IV weekly × 4 | NA | NA | + (Lympho-proliferative disease) | NA | NA | 27614471 |
| 15 | Case Report | 2017 | 1 | 1 | GLILD | 1g IV , 2x | MMF | / | + (GLILD, lymphadenopathy) | NA | NA | 27896807 |
| **16** | **Abstract** | **2018** | **1** | **1** | **GLILD** | **375 mg/m^2^ IV weekly × 4, 6-monthly** | **NA** | **/** | **+ (GLILD, ITP, splenic granulomas)** | **NA** | **Reduction in ALP** | **30602282** |
| 17 | Case Report | 2019 | 2 | 2 | GLILD | Different regimens | NA | / | + (GLILD, lymphadenopathies) | NA | NA | 31060706 |
| 18 | Case Report | 2019 | 1 | 1 | GLILD | 375 mg/m^2^ IV weekly × 4 | / | / | + (GLILD) | NA | NA | 31825316 |
| 19 | Original article | 2019 | 11 | 11 | GLILD | 375 mg/m^2^ IV weekly × 4 | NA | / | + (GLILD) | NA | NA | 30843876 |
| 20 | Case Report | 2019 | 1 | 1 | GLILD | 375 mg/m^2^ IV weekly, 6-monthly | AZA | / | + (GLILD) | NA | NA | 30984724 |
| 21 | Case Report | 2019 | 1 | 1 | GLILD | 375 mg/m^2^ IV weekly, 3-weekly | / | Infections (pneumococcal, herpes zoster reactivation) | + (GLILD, decrease in spleen size) | NA | NA | 30713498 |
| 22 | Case series | 2019 | 3 | 3 | GLILD | 375 mg/m^2^ IV weekly × 4, 6-monthly | / | / | + (GLILD) | 1 patient with 'NASH' | NA | 30408616 |
| 23 | Retrospective - Monocentre study | 2021 | 39 | 39 | GLILD | 375 mg/m^2^ IV weekly × 4, 6-monthly | AZA or MMF | Fatal septicemia (n = 1) | + (GLILD) | NRH (n = 7/39) | NA | 32745555 |
| 24 | Case Report | 2022 | 1 | 1 | GLILD | 375 mg/m^2^ IV weekly × 4, 3-monthly | / | / | + (GLILD) | NA | NA | 34718215 |

For data collection, we performed a comprehensive literature search in the electronic database MEDLINE with the following search terms: “common variable immunodeficiency”, “CVID” and “rituximab”. Relevant references within the retrieved articles were evaluated and included if relevant. Red *=* articles reporting on liver involvement, **Bold** = articles reporting on the effect of rituximab on liver involvement

NA = not applicable or not reported

* unknown whether patients with reported liver disease were treated with rituximab
